# Seropositivity for Pathogenic *Leptospira* in Dogs, Cats, and Horses at a Teaching Veterinary Hospital in Southern Chile

**DOI:** 10.3390/tropicalmed10090253

**Published:** 2025-09-03

**Authors:** Lucía Azócar-Aedo, Gloria Meniconi, Carolina Pino-Olguín, María Gallardo

**Affiliations:** 1 Facultad de Medicina Veterinaria, Escuela de Medicina Veterinaria, Universidad San Sebastián, Sede de la Patagonia, Puerto Montt 5480000, Chile; lucia.azocara@uss.cl (L.A.-A.); gloria.meniconi@uss.cl (G.M.); carolina.pino@uss.cl (C.P.-O.); 2 Facultad de Medicina y Ciencias de la Salud, Escuela de Medicina Veterinaria, Universidad Mayor, Campus Huechuraba, Santiago 8580000, Chile

**Keywords:** Leptospirosis, dogs, cats, horses, seropositivity, serogroups, antibodies, veterinary hospital, Chile

## Abstract

At a veterinary hospital in southern Chile, we conducted an epidemiological study involving domestic dogs, cats, and horses to determine the seropositivity for pathogenic *Leptospira* spp., identify the infecting serogroups, measure antibody titers, and characterize seropositive animals by sex and age. None of the sampled animals showed clinical signs of leptospirosis. The microscopic agglutination test, using a panel of eight serogroups, was used for diagnosis. The seropositivity was 36.5% (95% confidence interval [CI] = 24.5–48.4) in dogs, 12.9% (95% CI = 2.6–23.1) in cats, and 45.2% (95% CI = 30.1–60.2) in horses. Serological reactions were detected for the Tarassovi and Canicola serogroups in dogs, Sejroe, Canicola, Icterohaemorrhagiae, and Grippotyphosa in horses, and Tarassovi in cats. The most frequent antibody titers were 1:200 and 1:400 in dogs, 1:400 in cats, and 1:800 in horses. The distribution of seropositivity varied by sex and age across different animal species. The seropositivity for pathogenic *Leptospira* in dogs, cats, and horses attending a veterinary hospital underscores the role of domestic animals as sentinels for zoonotic diseases. This finding has implications for epidemiological surveillance systems in increasing awareness of seropositivity and establishing specific prevention measures to mitigate the risk of leptospirosis transmission.

## 1. Introduction

Leptospirosis is a reemerging zoonotic disease and a public health problem of global importance [1]. Despite its increasing prevalence in humans worldwide, epidemic potential, and considerable socioeconomic burden, it is frequently neglected and underdiagnosed [2,3,4]. Ranging from mild to severe, the clinical manifestations of leptospirosis can vary significantly in humans and animals [5]. The interconnectedness of human, animal, and environmental health emphasized in the “One Health” approach is fundamental for understanding leptospirosis [6].

The bacterium is primarily transmitted through contact with water or soil contaminated by urine and other fluids from infected animals [7,8]. Humans become infected upon contact when the microorganism penetrates through the skin or mucous membranes, especially via broken skin [9]. Veterinarians, farmers, agricultural workers, plumbing, sewer workers, and garbage collectors represent occupations and activities with a high risk of infection due to the likelihood of exposure to contaminated environments [7,10].

This disease has a complex epidemiology in which various domestic and wild animal species are involved [11]. Incidental hosts can suffer severe illness or even death, excreting leptospires only during the acute phase of the infection, while maintenance hosts act as reservoirs and may show mild or no symptoms but continuously excrete leptospires for months or their entire lifespan [12]. The knowledge of the infecting dose required to cause infections in a natural environment is based on studies with laboratory animal models, and infectious doses are likely to be considerably smaller, as extremely large doses are very unlikely to occur in nature [13].

Leptospirosis is a significant infectious disease in domestic dogs though often overlooked in the differential diagnoses of other pathologies [11]. The disease manifests as acute tubulointerstitial inflammation, nephritis, and/or liver dysfunction, but its impact is multisystemic [14]. Infected dogs can shed leptospires in their urine, posing a risk of leptospirosis to humans and animals, particularly in urban areas [15,16]. The prevalence of leptospirosis is higher in puppies, geriatric animals, and unvaccinated patients [14].

Domestic cats are incidental hosts of *Leptospira* serovars, which are prevalent in different animals [17]. Clinical signs in cats are typically nonspecific and can include polyuria, polydipsia, hematuria, uveitis, lethargy, anorexia, weight loss, vomiting, diarrhea, and inflammatory skin lesions. Cats kept in outdoor conditions have a higher risk of infection, as does the rural population due to contact with livestock [18]. Seropositivity in cats is also associated with the consumption of infected rodents [18,19].

In horses, leptospirosis can manifest as a subclinical disease [20] or cause intrauterine infection in pregnant mares, leading to abortions, neonatal diseases, or the birth of healthy but serologically positive foals. Furthermore, leptospirosis in horses is linked to recurrent uveitis, an autoimmune disease characterized by intraocular inflammation and pain following acute infection. If untreated, this condition can lead to blindness [21], making the disease a significant concern in equine clinical practice and a substantial economic burden [22].

Some epidemiological studies in Chile have determined seroprevalences in different domestic animal species, mainly in central and southern areas of the country, such as 69.7% in ruminants (cattle, sheep, and goats) [23], 21.3% and around 12.0% in canines [24,25], 15.6% and 25.2% in cats [26,27], 65.4% and 68.6% in horses [28,29], 5.7% in sheep [30], and 24.8% in goats [31]. To date, the study by Uribe et al. [23] has investigated seropositivity in various ruminant species, but few updated research has been conducted on multiple species, including both large and small animals in the same geographical location and having a cross-sectional design.

Dogs, cats, and horses can harbor circulating serogroups of *Leptospira* [32,33]. The epidemiological surveillance of transmissible diseases in hospitals and veterinary clinics involves detecting diseased individuals or those with clinical signs to establish the existence of the disease, identifying disease patterns, and formulating preventive measures [34]. This study arose from the need to generate epidemiological information on the exposure of multiple species of domestic animals to *Leptospira* spp. in a specific place where the interaction between animals and humans is continuous, such as a teaching veterinary medical hospital, involving both staff and students, as a way to illustrate the potential risk of infection. According to Rabinowitz et al. [35] these healthcare facilities serve as crucial points for identifying zoonotic agents in animals, providing evidence of the potential for transmission to humans.

This study aimed to determine the seropositivity for pathogenic *Leptospira* spp. in asymptomatic domestic dogs, cats, and horses at a veterinary hospital in southern Chile. The research also examined the frequency of serogroups and antibody titers and characterized seropositive animals by sex and age.

## 2. Materials and Methods

### 2.1. Study Design

This was a cross-sectional, quantitative, descriptive, and retrospective observational study [36].

### 2.2. Serological Samples

The study utilized a set of serological samples donated by the Clinical Laboratory at the Veterinary Hospital of the Facultad de Medicina Veterinaria, Universidad San Sebastián, Puerto Montt. These samples were collected between 2022 and 2024 from domestic dogs (*Canis familiaris*), domestic cats (*Felis catus*), and horses (*Equus caballus*) admitted to the hospital, but without clinical signs of leptospirosis and coming from different geographical areas of the Los Lagos region in southern Chile. All samples were from animals with a known guardian. In general, the canine and feline samples came from animals of urban and rural origin, with different maintenance conditions: primarily outdoor for dogs and mixed (indoor–outdoor) for cats. The equines were used for work, riding, and leisure.

The samples were obtained from leftover serum (0.5–2 mL) from blood tests, such as biochemical profiles, and were stored at −17 °C. The study utilized a total of 167 serological samples from three species: 63 canine samples, 62 feline samples, and 42 equine samples.

### 2.3. Microscopic Agglutination Test (MAT)

The serological samples were analyzed using the MAT test with a panel of eight serogroups belonging to *Leptospira interrogans*, *Leptospira borgpetersenii*, and *Leptospira kirchneri*. The strains were acquired from an international microbiology company and were cultured in the Infectious Diseases Laboratory of the Institute of Preventive Veterinary Medicine at Universidad Austral de Chile. The serogroups, serovars, and strains used are listed in Table 1.

### 2.4. Interpretation of MAT Results

The interpretation of MAT results varied by animal species.

(a) Dogs: For non-vaccinated animals, titers ≥ 1:100 were classified as positive [39]. For vaccinated dogs, the interpretation depended on the time elapsed since immunization. In dogs vaccinated 1 to 3 months before blood sample collection, titers ≥ 1:400 were considered positive for pathogenic *Leptospira* spp., while in those vaccinated between 3 to 12 months before sampling, titers ≥ 1:200 were classified as seropositive [25]. The vaccine commonly used in Chile for canine leptospirosis includes the Canicola and Icterohaemorrhagiae serovars. Vaccination data for patients were extracted from their clinical records.
(b) Cats: Titers ≥ 1:100 were classified as positive serological reactions to pathogenic *Leptospira* spp. [27].
(c) Horses: As vaccines for equine leptospirosis are not used in Chile [40], the presence of antibody titers ≥ 1:100 was considered indicative of seropositivity for the bacteria [41,42].

When a sample from any of the studied animal species presented equal titers against different serovars or serogroups, it was classified as coagglutinations [43].

### 2.5. Data Analysis

The seropositivity for pathogenic *Leptospira* in each animal species was calculated using the formula described by Beaglehole et al. [44] and Epidat 4.2 [45]. The 95% confidence intervals (95% CI) were also estimated [46]. The seropositivity for the serogroups and the frequency of antibody titers were visualized using graphs constructed with Microsoft Excel (from Microsoft Office 2016).

The age classification for dogs and cats was based on a modified version of the grouping provided by Vogt et al., Harvey et al., and Ricardo et al. [47,48,49]: junior (0–2 years for dogs and cats), adults (3–7 years for dogs and 3–10 years for cats), and senior (>7 years for dogs and >10 years for cats). For horses, the age categorization was as follows: young (<5 years), adult (≥5 and ≤10 years), mature (≥10 and ≤15 years), and old (>15 years) [50].

The Chi-square test with Yates correction [51] and EpiInfo version 6.04 were used to analyze differences between serogroup frequencies, antibody titers, and the sex and age distribution of seropositive animals in each species. Additionally, the association of sex and age with the presence of antibodies against pathogenic *Leptospira* for each animal species was assessed using the Fisher or Chi-square test with Statistix version 10 and EpiInfo version 6.04. A *p*-value < 0.05 was considered indicative of statistical significance for all analyses.

## 3. Results

### 3.1. Seropositivity for Pathogenic Leptospira

The seropositivity for pathogenic *Leptospira* was 36.5% (23/63, 95% CI = 24.5–48.4) in domestic dogs, 12.9% (8/62, 95% CI = 2.6–23.1) in cats, and 45.2% (19/42, 95% CI = 30.1–60.2) in horses. Notably, the differences between these proportions were not significant (*p* > 0.05).

### 3.2. Serogroups Most Frequently Diagnosed in Seropositive Animals

**Figure 1:**
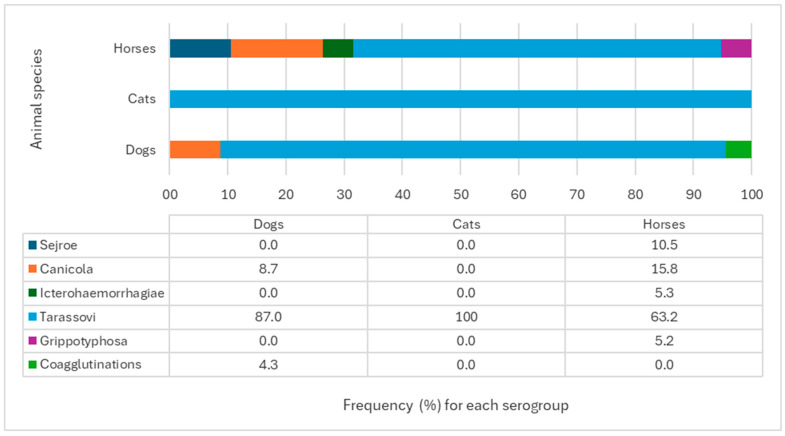
Seropositivity for pathogenic *Leptospira* serogroups among dogs, cats, and horses. Veterinary Clinical Hospital, Universidad San Sebastián, Puerto Montt, Chile.

In domestic dogs, seropositivity was detected for the Canicola (8.7%) and Tarassovi (87.0%) serogroups, with 4.3% showing coagglutinations (Figure 1). No significant differences were observed between these proportions (*p* > 0.05). In cats, all positive samples (100%) showed serological reactivity for the Tarassovi serogroup (Figure 1). In horses, seropositivity was found for the Sejroe (10.5%), Canicola (15.8%), Icterohaemorrhagiae (5.3%), Tarassovi (63.2%), and Grippotyphosa (5.3%) serogroups (Figure 1), with no significant differences observed between these frequencies (*p* > 0.05).

### 3.3. Antibody Titers Detected in Seropositive Animals

**Figure 2:**
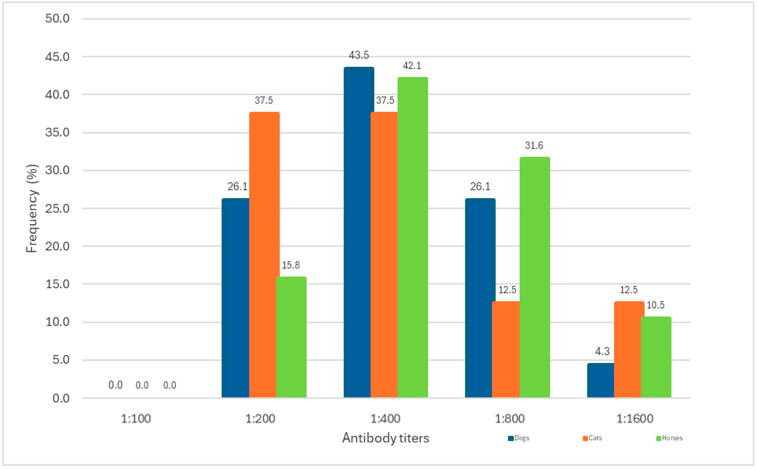
Distribution of antibody titers against pathogenic *Leptospira*, ordered by animal species. Veterinary Clinical Hospital, Universidad San Sebastián, Puerto Montt, Chile.

In domestic dogs, the highest frequencies of antibody titers were 1:400 (43.5%), 1:200 (26.1%), and 1:800 (26.1%), with 1:1600 titers being the least frequent (4.3%) (Figure 2). These differences were not statistically significant (*p* > 0.05).

In cats, antibody titers of 1:200 and 1:400 were equally frequent (37.5%), followed by titers of 1:800 and 1:1600 (12.5% each) (Figure 2). No statistically significant differences were observed (*p* > 0.05).

In horses, the antibody titers were predominantly 1:400 (42.1%) and 1:800 (31.6%), followed by 1:200 (15.8%) and 1:1600 (10.5%) (Figure 2). These differences were not statistically significant (*p* > 0.05).

Regarding the MAT results and vaccination in domestic dogs, no animals were detected positive for serovar Icterohaemorrhagiae (serogroup Icterohaemorrhagiae), and only three animals were recorded positive for serovar Canicola (serogroup Canicola). These animals were classified as positive for the presence of anti-*Leptospira* antibodies following the criteria indicated in the Materials and Methods Section regarding the time elapsed since vaccination until sampling and the diagnosis with the MAT.

Table 2 shows the number of positive animals for each antibody titer, according to the positive serogroups. The most frequently detected antibody titers were 1:200, 1:400, and 1:800 in dogs, cats, and horses, with the highest number of animals positive for serogroup Tarassovi. Only one positive sample with a titer of 1:100 was detected in serogroup Canicola in dogs. Titers of 1:1600 were less frequent for the three animal species analyzed and were found only for serogroup Tarassovi.

### 3.4. Characterization of Seropositive Animals by Sex and Age

In dogs, the seropositivity by age was as follows: junior (50%, 95% CI = 15.3–84.6), adult (23.1%, 95% CI = 0.1–45.9), senior (36.8%, 95% CI = 21.5–52.1), and age not specified (50.0%, 95% CI = 1.0–99.0). Regarding sex, seropositivity was 38.5% (95% CI = 19.7–57.1) in males and 35.1% (95% CI = 19.7–50.5) in females. No statistically significant differences were observed in the distribution by age and sex, and no association was found between seropositivity and sex or age (*p* > 0.05).

The seropositivity in cats according to age was as follows: junior (16.1%, 95% CI = 1.7–31.5), adult (13.6%, 95% CI = 0.0–27.9), and senior (10.0%, 95% CI = 0.0–28.5), with no statistically significant differences (*p* > 0.05). The seropositivity by sex was 14.3% (95% CI = 1.3–27.2) in males and 11.8% (95% CI = 0.9–22.5) in females, showing no statistically significant differences (*p* > 0.05). No association was detected between seropositivity and sex or age (*p* > 0.05)

In horses, the seropositivity by age was as follows: young (37.5%, 95% CI = 3.9–71.0), adults (25.0%, 95% CI = 0.5–49.5), mature (50.0%, 95% CI = 0.0–100), and old (100%, 95% CI = N/A), with 52.9% of cases having no recorded age. The differences between these values were not significant (*p* > 0.05). Regarding sex, the seropositivity was 39.1% (95% CI = 19.1–59.0) in males and 50.0% (95% CI = 26.9–73.1). No association was found between seropositivity and sex or age (*p* > 0.05).

## 4. Discussion

Due to the greater susceptibility of humans or environmental exposure, the presence of zoonotic diseases in animals can indicate a potential risk of transmission to humans, making the disease crucial from an epidemiological perspective [52]. This study detected seropositivity for pathogenic *Leptospira* in dogs, cats, and horses attending a veterinary hospital in southern Chile. The research identified the most frequent serogroups and antibody titers in seropositive animals and characterized these individuals by sex and age. The findings highlight the importance of domestic animals as sentinels for detecting positivity to zoonoses such as leptospirosis in a healthcare setting. The animals showed serological reactivity to the bacteria without displaying clinical signs or suspicion of the disease.

The seropositivity recorded for pathogenic *Leptospira* in canines (36.5%) was higher than previously reported in Chile, such as by Silva and Riedemann [43] (14.8%) in Valdivia, Tuemmers et al. [24] (21.3%) in Temuco, Lelu et al. [53] (25.1%) in the Los Ríos region, and Azócar-Aedo and Monti [27] in the Los Ríos and Los Lagos regions (12.3% in urban areas and 12.0% in rural areas). Conversely, higher seroprevalences have been reported in domestic dogs in some countries in the Americas, such as 75% in dogs sampled from five indigenous Kichwa communities living in the Yasuní National Park in the Ecuadorian Amazon basin [54], as well as lower frequencies of about 29.4% in Tennessee, USA [55]. A study in Brazil concluded that active serosurveys in dogs may provide early diagnosis and help for leptospirosis control, preventing human infections [56].

In felines, the seropositivity (12.9%) differed from previous epidemiological studies in Chile, which reported rates of 20.0% in Concepción [57], 1.8% in urban areas and 25.2% in rural areas of the Los Ríos and Los Lagos regions [27], and 15.6% in the Los Ríos region [26]. The seropositivity found is very similar to that reported in cats in Tennessee, USA (12.4%) [55], as well as to the global prevalence of approximately 11% determined in three systematic reviews and meta-analyses of published studies worldwide [19,58,59].

For horses, the prevalence (45.2%) was similar to that in a study in the Bío-Bío region (48.1%) [60] but differed from that reported by Troncoso et al. [61] (65.4%) in the La Araucanía region and Tadich et al. [62] in the central area of Chile (30.6% in draft horses and 23.3% in army animals). In other places, such as Tennessee, USA, high seroprevalences of 47.7% in horses have been reported [55], as well as in the state of Goiás, Brazil (61.6%) [42], and in tropical Ecuador, where the seroprevalence reached 100% [63]. Díaz et al. [32] concluded in a systematic review that horses are excellent sentinels to reveal circulating leptospiral serovars, proposing that this species be used in epidemiological surveillance studies at local and national levels.

The discrepancies in the reported prevalence rates of *Leptospira* antibodies in Chile in other epidemiological studies can be attributed to various factors, including the origin of the sampled animals and the diagnostic tests used. Different studies sampled animals from distinct regions that may have unique ecological and climatic conditions. Various studies also employed different diagnostic methods such as MAT [25], bacteriological culture, and polymerase chain reaction [26], or ELISA [57]. The Veterinary Clinical Hospital of the Universidad San Sebastián in Puerto Montt receives animals from dissimilar areas of the Los Lagos region in the south of the country. The climatic conditions and ecology of the geographical area are expected to influence the positivity, as the probability of leptospiral infection is increased by certain environmental conditions, such as warm temperatures in summer, humidity, neutral soil pH, and stagnant surface water [62,64]. The ecological characteristics in southern Chile, such as a temperate climate with high annual rainfall, promote bacterial survival in the environment, particularly in the spring and summer seasons [26]. The rainy climate increases soil humidity. Heavy rainfall and flooding have been shown to trigger leptospirosis outbreaks in certain areas across the world [4].

Comparing the seropositivity for pathogenic *Leptospira* calculated in this study with that from data from human cases is important. Some risk factors for the development of human leptospirosis, which were described in a recent systematic review, include contact with exposed animals (rats and pigs) and work with animals (slaughterhouse workers) [65]. Another study determined the multifactorial nature of leptospirosis, influenced by environmental conditions that fail to meet sanitation standards, along with community activities in the agricultural and livestock sectors [66]. In a study in Chile, animal variables (positive dogs and rodents and proximity to positive households) and dog counts, as well as proximity to *Leptospira*-contaminated water samples on farms, were significant drivers for the presentation of the disease in people [67]. Rabinowitz et al. [68] highlight that communication between human and animal health professionals, regarding diseases detected in sentinel animals, is often limited. The “One Health” concept emphasizes a collaborative approach, considering shared risks and promoting cooperation between animal and human health professionals. Scotch et al. [69] stress the importance of linking epidemiological surveillance data from animals and humans to identify priorities for building effective surveillance systems and to enable modeling and prediction of human risk from zoonotic diseases. This linking is particularly crucial for zoonotic diseases with epidemic potential, such as leptospirosis.

In the three animal species under study, the most prevalent serogroup was Tarassovi. Pigs and wild boars are considered the maintenance hosts for this serovar [70,71]. Antibodies for this serogroup has been detected in various wildlife species such as choroy parrots, bandurrias, Magellanic penguins, chilla foxes, pudus, and guiña in southern Chile [72]. In equines, a recent study also found seropositivity for the Tarassovi serogroup in the south of the country, possibly indicating a considerable frequency of presentation [29].

Dogs and horses in this study were also found seropositive for the Canicola serogroup. Domestic canines are considered the maintenance hosts for this serovar [37]. The Canicola serogroup positivity in dogs and equines may be attributed to contact with urine from reservoir dogs. This contact is facilitated by canine behavior, such as sniffing and licking other canines, and stray animals are more likely to be infected [73]. The existence of seropositivity to the Canicola serogroup in dogs is an interesting result that requires careful interpretation, and in this study, a classification of seropositivity for the Canicola and Icterohaemorrhagiae serovars based on the time from vaccination to blood sample collection was taken into account to differentiate whether the detected antibodies were vaccine related [25]. Only three dogs were positive for the Canicola serogroup, and none were positive for Icterohaemorrhagiae, leading to the conclusion that other serogroups have a higher prevalence in the sampled animals.

Equines in this study also showed positivity for the Sejroe, Icterohaemorrhagiae, and Grippotyphosa serogroups. This positivity demonstrates the presence of reactivity to various *Leptospira* serogroups associated with diverse maintenance hosts [38] and indicates that horses in this study are in contact with animals, potentially enabling interspecies transmission of *Leptospira*. This finding is consistent with previous reports of interspecies transmission in other animals in southern Chile [74].

In dogs, the highest frequencies of antibody titers were observed at 1:200 (26.1%), 1:800 (26.1%), and 1:400 (43.5%). Titers of 1:1600 occurred at a frequency of 4.3%. These results are consistent with the study by Silva and Riedemann [30] but differ from the results reported by Azócar-Aedo and Monti [25], who detected serological reactions with lower titers (1:100 and 1:200) in 80% of seropositive dogs. Notably, both of these studies were conducted in geographical areas close to those of the present study. In cats, antibody titers of 1:200 and 1:400 were equally frequent (37.5%), while titers of 1:800 and 1:1600 were at a frequency of 12.5% each. In the study by Azócar-Aedo et al. [11], out of seven *Leptospira*-positive animals, the majority presented titers of 1:100; only two cats demonstrated titers of 1:200, and one demonstrated a titer of 1:400. The information on the duration of leptospiral antibodies in cats is limited. However, studies suggest that infected animals may produce titers lower than 1:100 [17]. In horses, the most frequently observed antibody titers were 1:400 (42.1%) and 1:800 (31.6%), followed by 1:200 (15.8%) and 1:1600 (10.5%). In this species, titers of 1:200 to 1:400 are considered indicative of recent infections, while titers greater than 1:800 may indicate active infection [75]. In Chile, previous studies have reported varying titers. Troncoso et al. found titers of 1:100 and 1:400, while Tadich et al. [62] observed low antibody titers (1:100 and 1:200), with some high titers in urban working horses (1:400). Tapia [40] detected frequent serological reactions with titers of 1:200, followed by 1:100 and 1:400. Antibody titers ranged between 1:200 and 1:800, with titers of 1:1600 being less frequent, and the Tarassovi serogroup showing the highest titers. In leptospirosis, titers ≥ 1:400 in endemic areas and ≥1:100 in non-endemic areas are considered cases of infection when clinical signs of the disease are present [76], and other authors indicate that antibody titer values between 1:800 and 1:1600, with clinical signs, are evidence for a diagnosis of leptospirosis [7]. Therefore, this study included animals with evidence of exposure to the bacteria, which constitutes a public and animal health concern, necessitating the implementation of preventive measures.

In the present study, seropositivity was not found for serogroups included in the MAT panel, specifically Pomona (serovar Pomona), Autumnalis (serovar Autumnalis), and Australis (serovar Bratislava). The maintenance hosts for serovar Pomona are rodents [77], and seropositivity has been found in previous studies in Chile conducted on wild rodents in a rural area of the south of the country [78], as well as in dairy farms in southern Chile, where synanthropic species were determined to have a lower risk of infection than wildlife [79]. Another survey in Mediterranean Chile indicated that the Norwegian rat (*Rattus norvegicus*) was the species most infected with *Leptospira* spp. in agricultural areas [80]. In the case of Autumnalis, research in Chile has reported seropositivity in domestic dogs and cats [25,27], and the literature mentions that the reservoir is mice [77]. For the Australis serogroup, serovar Bratislava, seropositivity has been reported in horses in Chile [61], and among the maintenance hosts described for the serovar are rats, pigs, and horses [77].

In dogs, the seropositivity by age was 50% in juniors, 23.1% in adults, and 36.8% in seniors. Regarding sex, 38.5% of seropositivity was recorded in males and 35.1% in females. Other studies in Chile have found similar values in the sex distribution [24] and a greater number of serological reactants in young individuals (5 to 7 years). In contrast, Azócar-Aedo and Monti [25] reported a higher seroprevalence in females from urban environments and in males from rural areas.

In cats, the seropositivity by age was as follows: juniors 16.1%, adults 13.6%, and senior animals 10%. The seropositivity regarding sex was 14.3% in males and 11.8% in females. These results partially coincide with those reported by Azócar-Aedo et al. [27], who found that the majority of cats with antibodies against *Leptospira* spp. in the Los Ríos and Los Lagos regions of Chile were between 0.5 and 6 years old and were females.

In equines, the age and sex distribution of seropositive animals showed the following seropositivity rates: young 37.5%, adults 25.0%, mature 50%, old 100%, and 39.1% in males and 50.0% in females. The age-related results are consistent with the research by Troncoso et al. [61] who found relatively elevated seropositivity in equines aged 1–5 years, 6–10 years, and >15 years, with a higher prevalence in females. However, Tuemmers et al. [28] found no significant relationship between seropositivity and the variables studied.

In Chile, leptospirosis is reportable in humans but not in animals [81]. Some data on its incidence in people are as follows: 0.39 cases per 100,000 inhabitants in the Ñuble region, 0.10 cases per 100,000 people in the Valparaíso region, and 0.12 cases per 100,000 people in the Bío-Bío region [82]. Therefore, implementing a surveillance system based on the detection of seropositive animals in veterinary medical care establishments will provide updated epidemiological information. This data will help characterize clinical cases and animals with serological reactivity to pathogenic *Leptospira* spp., establishing the frequency of the pathology. Given the seropositivity of leptospirosis in dogs, cats, and horses found in the present study, adopting prevention strategies for leptospirosis and targeted surveillance is imperative due to the risk of transmission to humans. Veterinarians can help achieve this aim by providing adequate and understandable information to animal guardians.

The present study utilized donated samples collected for research purposes, making the calculation of a specific sample size impossible. As only one sample was available per animal, performing the MAT on paired serum samples to observe changes in antibody titers was not possible. Since we used serological samples to detect seropositivity, the results indicate exposure to the bacteria, which does not necessarily imply bacterial shedding into the environment. Future studies could consider direct diagnostic tests to detect the bacteria, for example, in urine samples. Furthermore, none of the animals presented clinical signs of leptospirosis, making it difficult to link seropositivity with disease impact. Other studies to determine factors possibly associated with seropositivity, taking into account demographic characteristics, maintenance or lifestyle of the animals, and environmental variables would be interesting.

The study was conducted in a veterinary hospital, a place which could differ in exposure risk compared to the broader domestic animal population. However, the detection of exposure and different levels of anti-*Leptospira* antibodies indicates the circulation of the bacteria in the environment, which constitutes a concern for public and animal health. Despite these limitations, the study achieved its specific objectives, demonstrating seropositivity in animals attending a medical care facility and the relevance of domestic animals admitted to veterinary hospitals as sentinels for detecting serological reactivity and antibodies for zoonotic diseases such as leptospirosis, as well as the latent risk of transmission of the infection to people.

## 5. Conclusions

This study at the Veterinary Clinical Hospital of the Universidad San Sebastián, Puerto Montt, in southern Chile, found that domestic dogs, cats, and horses were serologically positive for pathogenic *Leptospira*. The seropositivity values differed among animal species. The Tarassovi serogroup was the most frequently detected in seropositive animals. Antibody titers ranged between 1:200 and 1:1600, varying by species. The distribution of positive animals by sex and age also differed by species. The seropositivity for pathogenic *Leptospira* in dogs, cats, and horses highlights the role of domestic animals as sentinels for detecting zoonotic diseases in animal healthcare settings. This information is crucial for epidemiological surveillance systems to monitor and characterize the frequency of the pathology, ultimately informing specific prevention measures.

## Figures and Tables

**Table 1:** Serogroups, serovars and strains of the MAT panel.

Serogroups	Serovars	Strain
Pomona	Pomona	Pomona
Canicola	Canicola	Hond Utrech IV
Icterohaemorrhagiae	Icterohaemorrhagiae	Icterohaemorrhagiae
Autumnalis	Autumnalis	Akiyami A
Australis	Bratislava	Jez Bratislava
Sejroe	Hardjo	Ar
Tarassovi	Tarassovi	Perepelitsin
Grippotyphosa	Grippotyphosa	Sag

The MAT followed the protocol described by Faine and WHO/ILS [37,38]. The positive samples from the screening phase were titrated, using antibody titers of 1:100, 1:200, 1:400, 1:800, and 1:1600.

**Table 2:** Frequency of antibody titers (number of positive animals), according to positive serovars in the animal species included in the study. Veterinary Clinical Hospital, Universidad San Sebastián, Puerto Montt, Chile.

		Antibody Titers					
Animal Species	Serogroups	1:100	1:200	1:400	1:800	1:1600	Total
Dogs	Canicola	1	0	1	0	0	2
	Tarassovi	0	5	8	6	1	20
	Canicola-Tarassovi	0	0	1	0	0	1
Cats	Tarassovi	0	3	3	1	1	8
Horses	Canicola	0	1	2	0	0	3
	Tarassovi	0	2	4	4	2	12
	Grippotyphosa	0	0	1	0	0	1
	Sejroe	0	0	1	1	0	2
	Icterohaemorrhagiae	0	0	0	1	0	1

## Data Availability

The original contributions presented in this study are included in the article. Further inquiries can be directed to the corresponding author.

## Abbreviations

The following abbreviations are used in this manuscript:

MAT	microscopic agglutination test
95% CI	95% Confidence Interval
